# RemoteHealthConnect: Innovating patient monitoring with wearable technology and custom visualization

**DOI:** 10.1177/20552076241300748

**Published:** 2024-12-10

**Authors:** Sasipriya Arun, Edward R. Sykes, Syed Tanbeer

**Affiliations:** 1 Centre for Applied AI, 6166 Sheridan College, Oakville, ON, Canada; 2 AI Lab, School of Computer Science, 3653 University of Guelph, Guelph, ON, Canada; 3 Faculty of Applied Science and Technology, 6166 Sheridan College, Oakville, ON, Canada

**Keywords:** Mobile health (mHealth), e-Health, healthcare IT, web-based healthcare, remote patient monitoring, continuous vital monitoring, medical data visualization

## Abstract

**Objective:**

This paper introduces *RemoteHealthConnect*, a novel web-based healthcare system designed to enable healthcare professionals to monitor patients remotely with enhanced efficacy. Central to our system is its integration with the Vitaliti™ wearable, equipped with biosensors for real-time vital signs monitoring. *RemoteHealthConnect* distinguishes itself by offering advanced, custom visualizations for interactive engagement with medical data, facilitating rapid clinical decision-making through intuitive access to vital signs and trends. The primary research question we sought to answer was: ‘Which design of vital sign visualizations is most effective in improving intuitive and rapid understanding for healthcare practitioners?’

**Methods:**

An iterative agile/SCRUM methodology was employed in the design and development of *RemoteHealthConnect*. We describe the architectural design of our web-based application, data visualization techniques, and user interface design. A user interface/user experience (UI/UX) study was conducted to assess the efficacy of our system.

**Results:**

The usability study revealed the system's capacity to translate complex bedside data into accessible, real-world visualizations, promoting efficient pattern recognition and anomaly detection. This is crucial for enhancing clinician performance, regardless of the patient's location. The paper further details a usability study involving healthcare practitioners to ascertain *RemoteHealthConnect's* efficacy. The System Usability Scale (SUS) assessment yielded a score of 71.5, indicating high usability. This score is significant, positioning our system above the average usability threshold for healthcare technologies, and suggesting it as a valuable tool for remote patient monitoring.

**Conclusion:**

Our web-based healthcare system and findings from the usability study contribute to the domains of Mobile Health (mHealth) and e-Health by advancing remote monitoring capabilities and offering a promising avenue for healthcare IT to improve patient care and clinician workflow.

## Introduction

The remote patient monitoring (RPM) sector, a critical component of mobile health (mHealth), has seen substantial growth, valued at 5.2 billion US dollars in 2023.^1,2,3^ With an anticipated compound annual growth rate of 18.6% from 2024 to 2030, the RPM industry is expanding due to aging populations, the versatility of RPM systems beyond acute care, and the optimization of hospital resource allocation.^3,4^

One of the pivotal challenges in RPM and mHealth is the effective visualization of patient vital data for medical decision-making.^5^ Traditional chart-based and tabular data representations often fall short in conveying complex data intuitively. There is a pressing need for healthcare professionals to access real-time data, identify trends, and review both short-term and long-term historical data efficiently.

**Figure 1.**
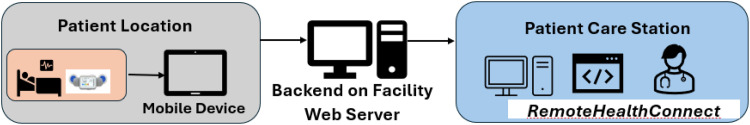
Overview of the RemoteHealthConnect system.

Addressing these challenges, this paper presents *RemoteHealthConnect*, the culmination of a multi-year project aimed at developing innovative visualizations for medical practitioners. Utilizing the Vitaliti^TM^ Continuous Patient Monitoring (CPM) wearable developed by Cloud DX,^6^ *RemoteHealthConnect* provides a comprehensive solution for the real-time monitoring of vital signs and physiological signals. This research highlights the significance of custom visualizations in promoting the early detection of health issues, optimizing RPM, and enhancing overall healthcare management. Figure 1 offers a high-level overview of the *RemoteHealthConnect* system, illustrating the seamless integration between the mobile device and Vitaliti^TM^ wearable situated at the patient's location (left component), the backend services (middle component), and the Patient Care Station (right component). At the Patient Care Station, health professionals interact with *RemoteHealthConnect* to access patient data, trends, and visualizations, enabling informed medical decision-making.

**Figure 2.**
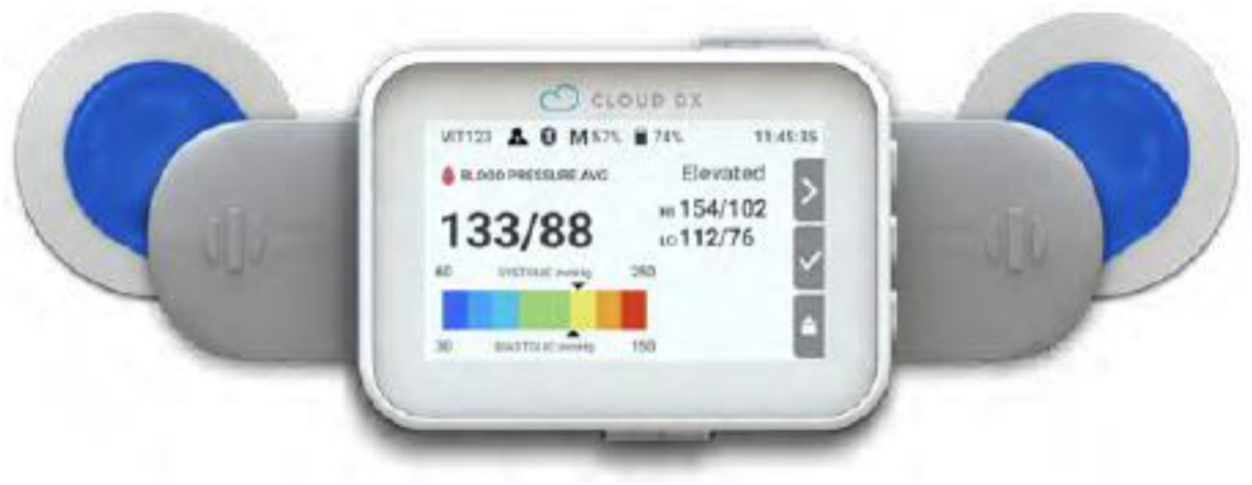
Cloud DX's Vitaliti^TM^ continuous patient monitoring wearable.

The *RemoteHealthConnect* system integrates seamlessly with the Vitaliti^TM^ CPM wearable, designed and developed by Cloud DX.^6,7^ Distinguished by its innovative design, the Vitaliti^TM^ wearable concurrently monitors five critical vital signs: blood pressure (BP), heart rate (HR), body temperature, blood oxygen saturation (SpO_2_), and respiratory rate (Resp Rate). Moreover, it captures and transmits raw physiological signals, including electrocardiogram (ECG), photoplethysmogram (PPG), and accelerometer data, all in real-time. This capability ensures that healthcare professionals have access to a comprehensive set of vital data necessary for effective remote monitoring, through a single, streamlined device. Figure 2 showcases the Vitaliti^TM^ wearable.

This research demonstrates that custom visualizations of vital data enable early detection of health issues, effective RPM, and improved healthcare management. The principal contributions of this study include:

- Development of customized dashboards tailored to address specific research questions.
- Implementation of customizable data range selections for vital sign charts, enabling detailed historical data analysis.
- Innovative design of vital data charts, including a mode selection feature for user preference (including light vs. dark mode).
- A radial chart component that displays multiple vital signs data over a 24-h period, updated hourly.
- Application of color coding to differentiate vital data values and their respective ranges for enhanced interpretability.

The structure of this paper is as follows: First section is Introduction. Literature Review section delves into the background of wearable technology and CPM systems, examining contemporary data visualization strategies within existing web-based health applications. Methods section details the architecture, design, methodology, and functionality of the *RemoteHealthConnect* system, including a discussion on its benefits and limitations. Results section outlines the results from a mixed-methods survey, incorporating both qualitative and quantitative data from usability studies. Discussion section discusses these findings, offering insights and implications for practice. Finally, Conclusion section concludes the paper, summarizing the study's outcomes and suggesting directions for future research and potential enhancements to the system.

## Literature review

This literature review examines critical domains pertinent to the development and application of *RemoteHealthConnect*, focusing on: (a) the evolution and impact of wearable technology in continuous vital sign monitoring; (b) the architecture and effectiveness of remote monitoring systems in healthcare; (c) the role of data visualization techniques in facilitating accurate and swift decision-making by healthcare professionals; and (d) a comparative analysis of existing web-based health applications, highlighting their features, limitations, and contributions to remote patient care.

### Wearable technology and continuous vital monitoring

The continuous monitoring of vital signs represents a pivotal component of contemporary healthcare. Wearable technologies, such as smartwatches and fitness trackers, have emerged as popular tools for the ongoing observation of essential health metrics, including HR, BP, and oxygen saturation. Research has shown that both patients and healthcare professionals in general hospital wards positively receive these technologies.^8^ The integration of continuous vital signs monitoring beyond the confines of critical care settings has been suggested to potentially enhance patient outcomes and reduce healthcare costs.^1,8–10^

This form of monitoring is vital for promptly detecting abnormal vital sign readings in patients at risk of clinical deterioration. Fluctuations in vital signs are recognized as sensitive indicators of potential clinical decline, heralding severe adverse events such as heart attacks or even mortality.^11^

A systematic review evaluating various wearable devices has highlighted the importance of comfort, accuracy, and the secure transmission of data.^12^ Most of these devices are capable of monitoring between two to five vital signs. Our research utilizes the Vitaliti^TM^ device, which aligns with the design recommendations identified in the review.^13^ The Vitaliti^TM^ device is distinguished by its ability to remotely and continuously measure five vital signs, in addition to capturing physiological signals such as ECG, PPG, and accelerometer data, thus addressing the critical aspects of wearable technology in healthcare.^13^

### Remote patient monitoring systems

RPM represents a significant segment of telehealth, leveraging digital tools and devices to gather, analyze, and store health data generated by patients outside traditional clinical environments. According to,^14^ RPM is propelled by technological advancements that facilitate the acquisition of patient information via sensors, wearables, smartphones, and cameras. This methodology is particularly noted for its effectiveness in identifying early signs of chronic condition decompensation, averting hospital admissions, and enabling constant health surveillance.^14^

RPM stands out for its capacity to monitor individuals with mobility challenges, offer timely interventions, and expand healthcare access to underserved regions.^14^ It has proven particularly beneficial in managing conditions such as diabetes, heart failure, and chronic obstructive pulmonary disease (COPD), where its application has been linked to reduced mortality rates and decreased hospitalizations. From the viewpoint of patients, RPM is valued for enhancing self-care, promoting overall health and well-being, and improving communication with healthcare professionals. As demonstrated in^15^ sensors in remote monitoring systems are often tailored to specific health conditions, leading to potential integration challenges and processing overhead when multiple devices are involved.^15^ Research highlighted in Ref.^16^ evaluates various RPM systems, classifying them according to their applicability to medical conditions such as cardiovascular and respiratory diseases, fall detection and mobility disorders, neurological and mental health issues, diabetes, and other health applications. This evaluation suggests a gap in the availability of a unified system capable of monitoring multiple health conditions simultaneously.

In response, our *RemoteHealthConnect* system is engineered to offer a universal and adaptable solution for medical professionals. It enables the real-time monitoring of multiple vital signs and features customizable dashboards tailored to specific health conditions, bridging the identified gap in current remote monitoring technologies.

### Vital visualization for clinical decisions

Remote health monitoring, facilitated by sensor technology, encounters the challenge of managing large, complex, and often unstructured health data from diverse sources. The dynamic and sometimes incomplete nature of this data complicates effective utilization. Data visualization therefore becomes critical in remote monitoring systems, offering healthcare professionals a coherent view of a patient's health status, thereby enabling smart, cost-effective decision-making.^17^ User-centered visualizations that present essential information are key to this process.^18^

Techniques such as dashboards, charts, graphs, and maps have been proposed for Internet of Medical Things systems to address these challenges.^19–21^ A notable study^22^ introduces a dashboard for visualizing data from distributed sensors across multiple patients treated in the same facility, allowing for the real-time monitoring and analysis of data. This underscores the necessity for customizable systems capable of monitoring various health conditions, with enhancements like 24-h data views to aid diagnosis. However, this study did not explore the usability or user satisfaction of their proposed solution.

An IoT framework for automated health analysis and management, leveraging cloud-based data processing, is discussed in.^23^ It outlines a comprehensive system comprising sensor-based data collection, mobile device data transfer, cloud processing and analysis, and mobile application feedback. This framework aims to support chronic disease management, health and wellness monitoring, and patient remote monitoring, highlighting the cloud's role in enabling real-time analysis and personalized healthcare services. The specifics of the algorithms for data analysis or the sensors for data collection were not detailed, with the study suggesting potential benefits including increased patient engagement and reduced healthcare costs.

The work in^24^ presents a graph-based visualization method for sensitive medical data, utilizing graph theory for a clearer, more comprehensive representation of medical data. This approach facilitates easier identification of significant patterns, particularly demonstrated through a case study on HR variability. This novel visualization method promises enhanced precision in medical data analysis, though its applicability to other medical data types remains unexplored.

Interactive visualizations, by enabling users to seamlessly navigate between views, provide a contextual and detailed understanding of data, essential for investigating specific queries or hypotheses. Thoughtfully designed interactive tools are indispensable for maintaining context, identifying patterns, and supporting diverse analytical tasks.^25^ In contrast, non-interactive visualizations may not adequately represent complex data dynamics, being more suited to static information dissemination in printed reports or emails.

The importance of data visualization in healthcare cannot be overstated, providing numerous advantages that significantly improve patient care and healthcare delivery.^25^ Key benefits include:

Enhancement of Patient Care: Real-time visualization of patient health data is fundamental in elevating care quality. It equips healthcare providers with the critical information needed to make informed clinical decisions tailored to the patient's current health status.^26^

Disease Trend Analysis and Pattern Recognition: Effective data visualization facilitates the identification of disease trends and patterns within specific vital sign data. This capability is essential for informed healthcare planning and intervention, leading to improved health outcomes. Tukey et al.^27^ highlight the use of exploratory data analysis (EDA) to detect trends, patterns, and outliers in health data, proposing it as a critical, iterative approach where graphical displays play a central role in hypothesis generation.^27^

Simplification of Data Presentation: Visualization techniques in healthcare distill complex data into formats that are accessible and comprehensible to a broad audience. This simplification is particularly crucial when explaining medical data to patients from diverse backgrounds, ensuring that information on disease prevalence and risk factors is easily understandable.^28^

Acceleration of Healthcare Provider Performance: Data visualization significantly boosts healthcare provider efficiency by enabling swift clinical decision-making. This acceleration not only positively affects patient prognosis but also diminishes care delays, contributing to the overall efficacy of healthcare organizations and leading to superior patient outcomes.^29^

Clinical Dashboards: Effective clinical dashboards incorporate elements such as bar charts, tables, icons, symbols, images, and color coding for organized information presentation. Features including radio buttons, expand/collapse options, and multi-view support are crucial for dashboard customization, catering to the varied needs of different users and enhancing usability.^30^

### Comparison of mobile and web-based health applications

The market has seen a rise in for-profit companies developing systems for remote monitoring systems.^31,32^ Below are some of relevant commercially available products, each designed to monitor specific vitals related to health conditions:

Dexcom: This Continuous Glucose Monitoring system features a connected mobile app providing real-time glucose insights, trends, and results on a single screen.^33^ The trend screen, color-coded for easy interpretation, displays the percentage of readings in normal, high, very high, low, and very low ranges. Its primary limitation is the focus solely on blood glucose level monitoring.

One Pulse LTE-M Smartwatch: A comprehensive health solution for the chronically ill and elderly, this smartwatch transmits data on HR, location, movement, and sleep via Bluetooth.^34^ However, it lacks features for remote monitoring by medical practitioners.^34^

eCareCoordinator: A telehealth software platform that allows clinicians to monitor patients’ vital signs remotely.^35^ It provides a portal for virtual monitoring and patient health management but does not offer trend visualization over time, which could help clinicians identify patterns or anomalies more efficiently.

While some of these products are designed to interface with external health monitoring devices, the data visualization capabilities within these applications often remain basic and underdeveloped.^36^ Most have not undergone verification or validation by medical practitioners, leaving their practical utility in question. Several health monitoring applications have emerged from research endeavors, demonstrating innovative approaches and technologies.

Real-Time Six-parameter Vital-Sign Monitoring in Ref.^37^ has introduced a web-based application for monitoring six vital parameters in real time, utilizing color coding to distinguish between them on a single page. This application's vital sign monitoring page shows ECG and PPG data in graphical formats. Enhancements to this design like data trends and customizable visualization designs could further benefit medical practitioners. The color coding is only used to distinguish the vital values not helpful in identifying if the values given fall in the normal, warning, or danger range. Also, this application focuses only on heart monitoring, whereas an application that can support dashboards for a wide range of health conditions and including comprehensive visualizations for all vital data can be more beneficial.

### Summary of Literature Review

This review of existing health monitoring systems has highlighted several shortcomings in the current landscape. Predominantly, these systems lack support for customizable visualizations, are designed to monitor specific health conditions with limited vital signals, and offer minimal user customization, including historical data viewing options. Based on these observations, we have pinpointed key gaps that our system aims to address, guiding the requirements (R) for its design:

R1: Audience-Centric Design: Our system will feature visualizations crafted with the end-user in mind, offering physicians dashboards with customizable components that are tailored to specific clinical problems.

R2: Versatile Visualization Selection with Custom Time Frames: Recognizing the varied nature of health data, our visualizations will accommodate different types, such as utilizing line or bar charts for trend analysis and heatmaps for vital sign ranges. These visualizations will include scrollable, custom time selection options.

R4: Comprehensive 24-h Vital Data Display: A specialized component will be introduced to provide hourly updates on average vital data values over a 24-h period, accommodating multiple vital signs.

R5: Insightful Data Highlighting: Key insights will be accentuated using color coding, tooltips, or annotations to draw attention to important data points within the visualizations.

R6: Clear and Simple Data Representation: We will ensure that visualizations are labeled precisely and clearly, accurately reflecting the data presented, to facilitate ease of understanding.

These identified gaps and corresponding requirements form the foundation of our system's design strategy, aiming to enhance the functionality and user experience of health monitoring systems.

## Methods

This section presents the design and evaluation of our system. The nature of this study involved the following:

1. Architectural design of our web-based application,
2. Data Visualization Techniques,
3. User Interface Design, and
4. Usability Study.

The entire study took place from May 2021 to May 2024 at the Centre for Applied AI (formerly, Centre for Mobile Innovation) at Sheridan College, Institute of Technology and Advanced Learning, Oakville, Ontario, Canada. Much of that time was spent on phases 1–4 above. The usability study took place from January 2024 to May 2024.

### Architectural design of our web-based application

**Figure 3.**
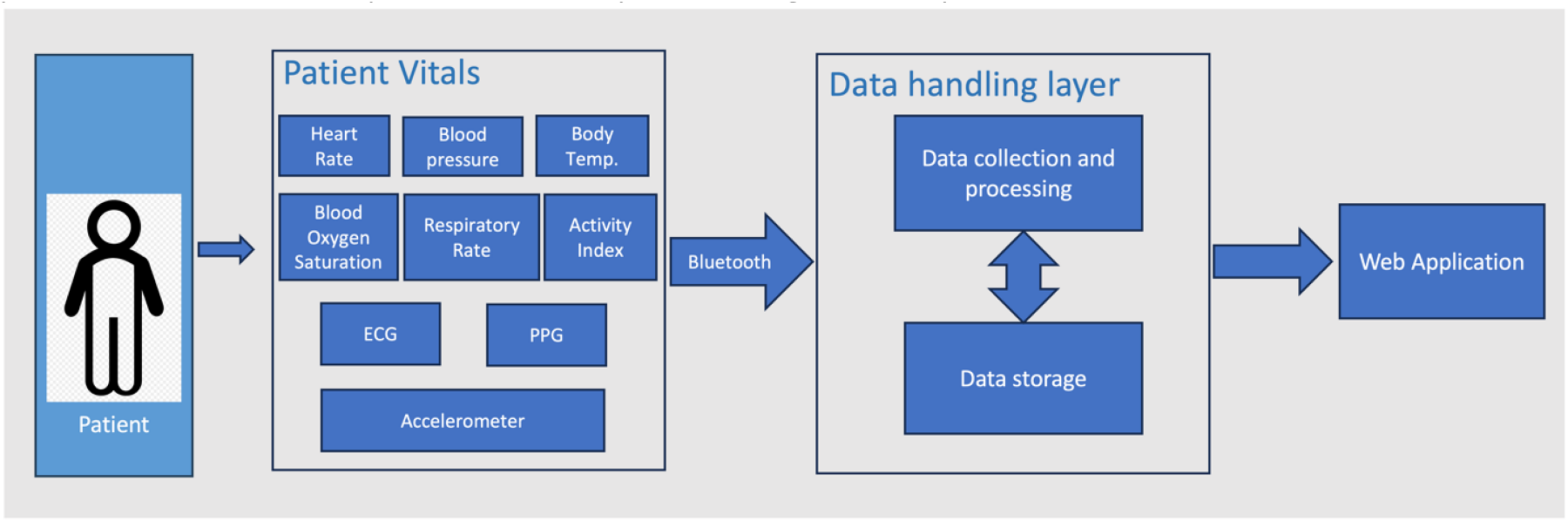
High-level architecture of the RemoteHealthConnect system.

The high-level architectural framework of *RemoteHealthConnect* is presented in Figure 3. Implemented as a multi-tier application, the architecture integrates the Vitaliti^TM^ wearable device for continuous real-time monitoring of patient vitals such as HR, BP, respiratory rate, body temperature, and SpO2, alongside physiological signals like ECG, PPG, and accelerometer data. This device connects via Bluetooth to a bedside application that serves as the Data Handling Layer, responsible for receiving, processing, and structurally storing vital data and physiological signals. Both patients and caregivers have access to vital information through this bedside application.

The web application liaises with the Data Handling Layer to fetch both real-time and stored data, incorporating various user interface visualization components. These components allow medical practitioners to monitor patients remotely, facilitating efficient patient care.

### Data visualization components

The design of dashboards and charts within our web application equips medical practitioners with critical information necessary for the effective remote monitoring of patients. These visualization tools act as a comprehensive gateway to patient health, offering insights and vital trends in real time. Designed for intuitiveness and user-friendliness, the interface ensures that medical practitioners of all technical abilities can effortlessly navigate and interpret the presented data. This approach not only enhances the decision-making process but also optimizes the efficiency of patient monitoring.

**Figure 4.**
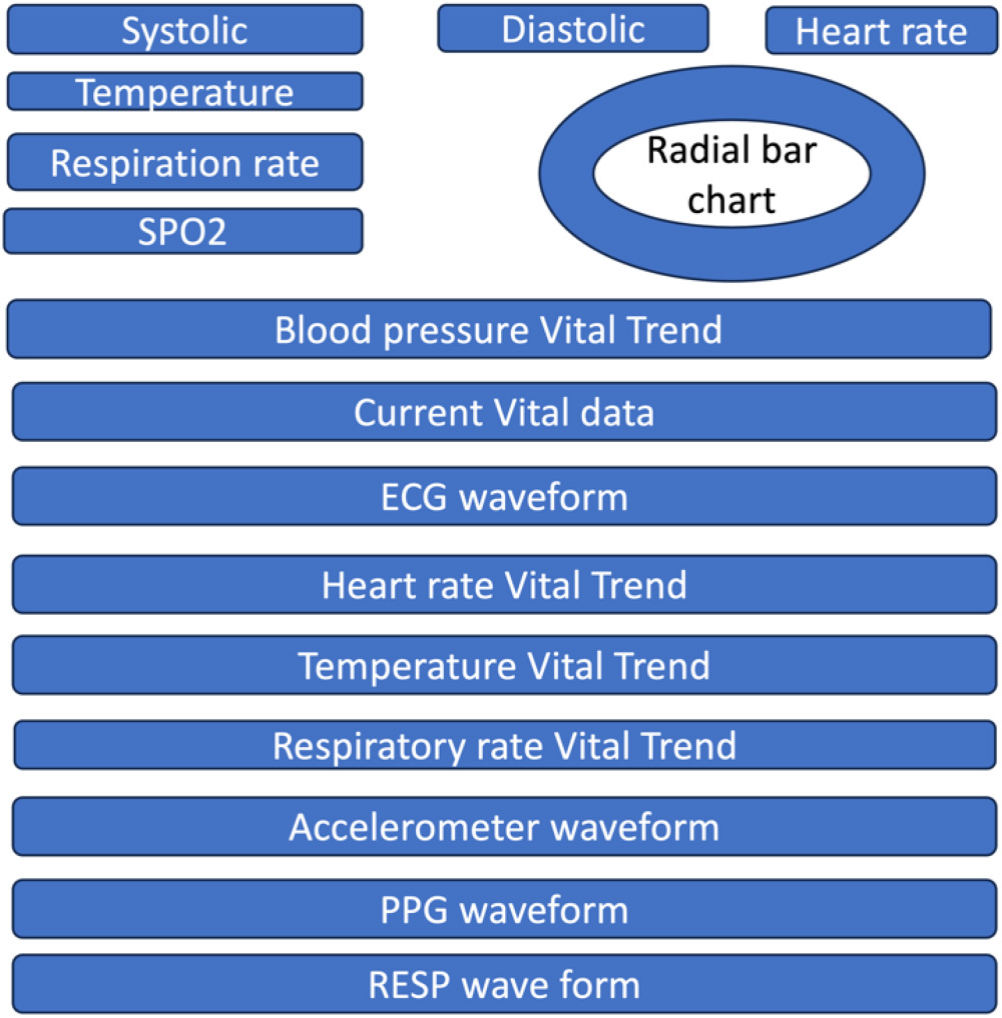
Default dashboard vital layout for RemoteHealthConnect.

*RemoteHealthConnect* incorporates several user interface components designed for intuitive and efficient display of health data.^37^ Designed to present crucial health information at a glance, dashboards of *RemoteHealthConnect* enable healthcare providers to gain a comprehensive and intuitive understanding of patient health status. The requirement R1 was implemented through:

- Clinician Dashboard: Features include patient personal details, symptom reports, medication summaries, health history, and allergy records.
- Condition-Specific Tabs: Offer specialized views for monitoring different health conditions such as Cardiovascular, Activity, Respiratory, etc., catering to specific monitoring needs. The design layout for the default tab is presented in Figure 4. The below section explains each of the component that is present in the layout namely Vital components which displays the real time values for the five vitals monitored, Vital trends designs for the vitals, Radial bar chart which displays 24-h data and the real time physiological signals-ECG, PPG, Accelerator and Respiratory waveform.

#### Vital components

**Figure 5.**
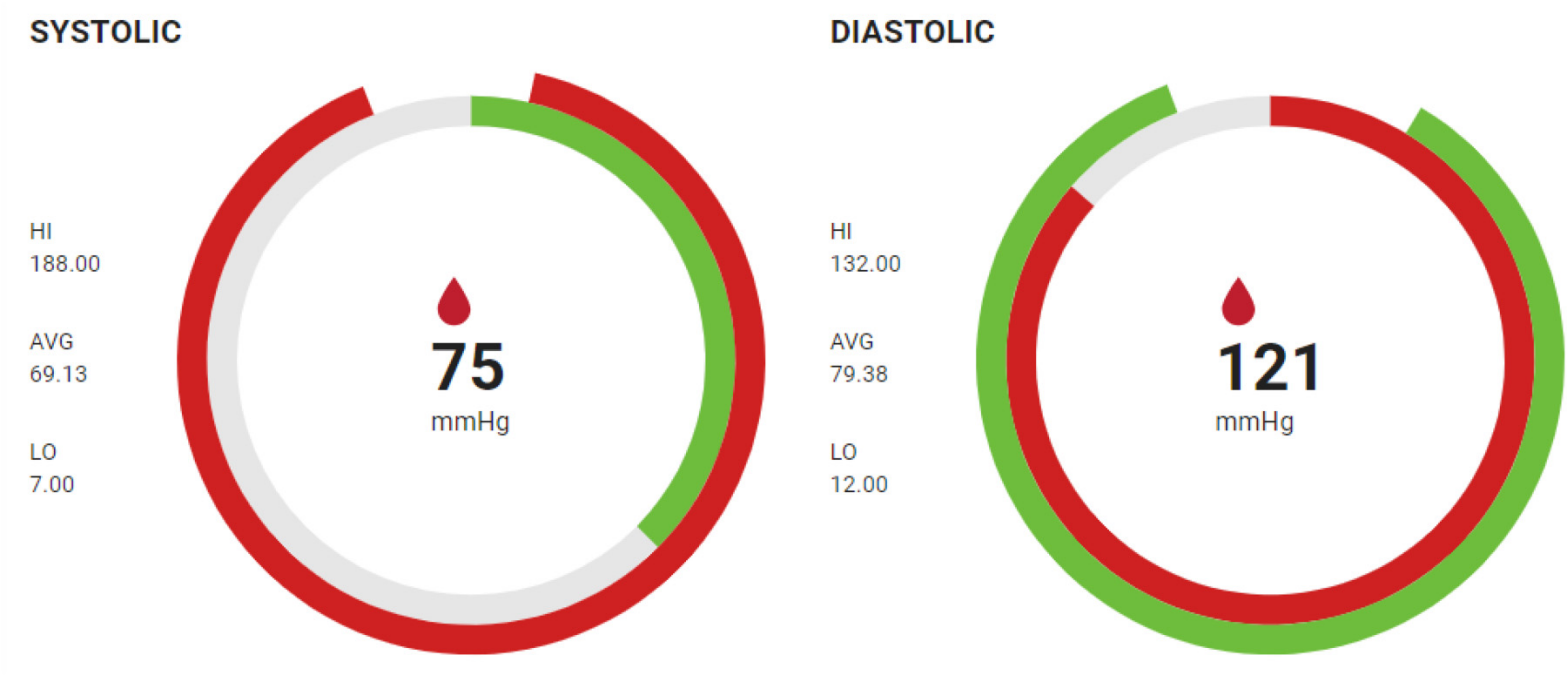
Vital component showing blood pressure vital—both systolic and diastolic.

The vitals component is used to display the real time vital values of the five vitals, namely, BP Systolic and Diastolic, HR, temperature, respiration rate, and SPO_2_ that are monitored in the system. They use radial charts and offer an intuitive and space-efficient method of visualization. As illustrated in Figure 5, the vital card for BP—specifically Systolic and Diastolic readings—demonstrates the innovative use of these charts. In the Systolic chart, the inner arc represents real-time data for the vital sign, spanning from 0 to the current value, which in this case is 75, color-coded in green to indicate that it falls within the normal range. Likewise, the outer arc visualizes high, low, and average values observed over the past 15 min, with the chart beginning at the lowest recorded value and extending to the highest. The color coding of this arc is determined by the 15-min average, which, is in the danger range for Systolic BP, is accordingly indicated.

This design approach ensures that clinicians can quickly grasp the patient's current vital status and historical trend within a concise visual space, enhancing the decision-making process.

#### Raw waveform components

The *RemoteHealthConnect* system is equipped to display real-time raw waveform data directly from the monitoring device. The *ECG* waveform captures and displays the electrical activity of the heart from multiple perspectives (Leads 1–4). The *PPG (Photoplethysmography)* waveform offers insights into blood volume changes by measuring light absorption or reflection. The *Accelerometer* data tracks and visualizes patient movement data, and the *Respiratory* waveform illustrates the patient's breathing pattern and rate.

**Figure 6.**
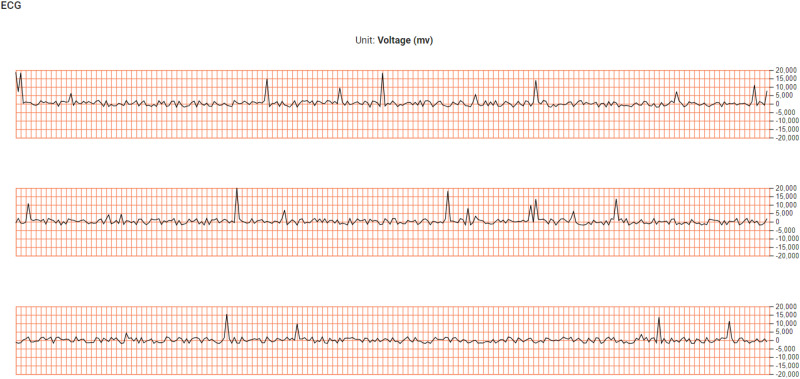
ECG visualization for leads 1–3 (facilitated by the Vitaliti^TM^ wearable).

These signals are presented in real time to provide healthcare practitioners with a comprehensive overview of the patient's physiological state. Figure 6 exemplifies the visualization of ECG data across Leads 1–3, showcasing the system's capability to deliver critical information with precision and clarity (In comparison to the system in literature review which has two leads for ECG^38^). This design not only facilitates immediate interpretation of vital physiological signals but also supports rapid clinical decision-making by offering detailed insights into the patient's current health status.

#### Vital trend component

**Figure 7.**
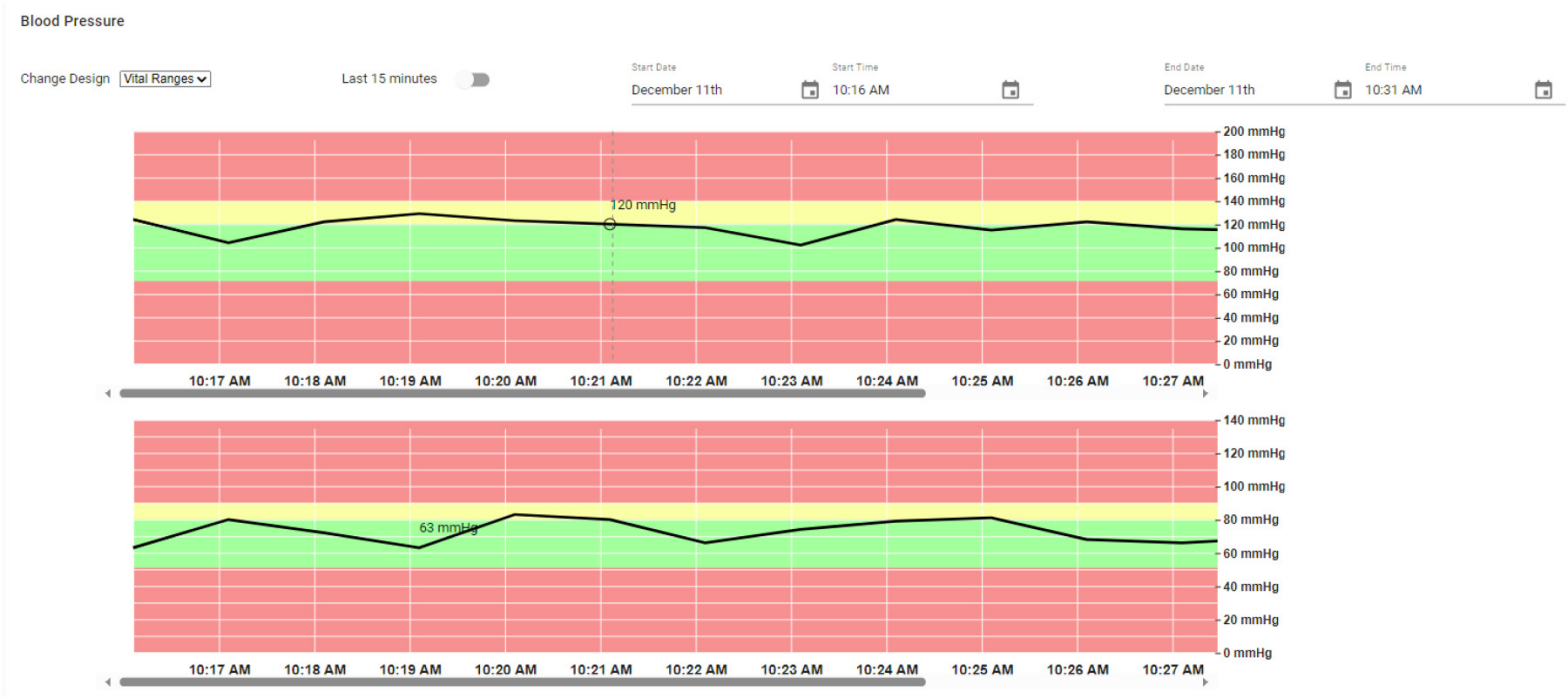
Design 1: color coded bands for vital ranges.

The vital trend component stands out as a particularly versatile tool, designed to meet the specific needs of medical practitioners. It allows users to select any custom date/time range for visualizing vital sign data, directly addressing system requirement R2. Moreover, this component offers customizable design options, enabling practitioners to tailor the visualization to their preferences. One of the design options named, *Vital Ranges*, is demonstrated in Figure 7 for BP vital, while the supplemental section includes a few more effective design options. As shown in the figure the visualization background of Vital Ranges design is color coded to intuitively indicate the normal (green), warning (yellow), and danger (red) ranges for the vital sign being monitored. This design allows users to easily understand the status of vital sign data within its respective range. By default, this component displays the last 15 min of real-time vital data. However, when the user opts out of the default setting, they can specify a custom time/date range to review historical data, enhancing the flexibility and utility of the system for ongoing patient care and analysis.

#### Radial bar chart component

**Figure 8.**
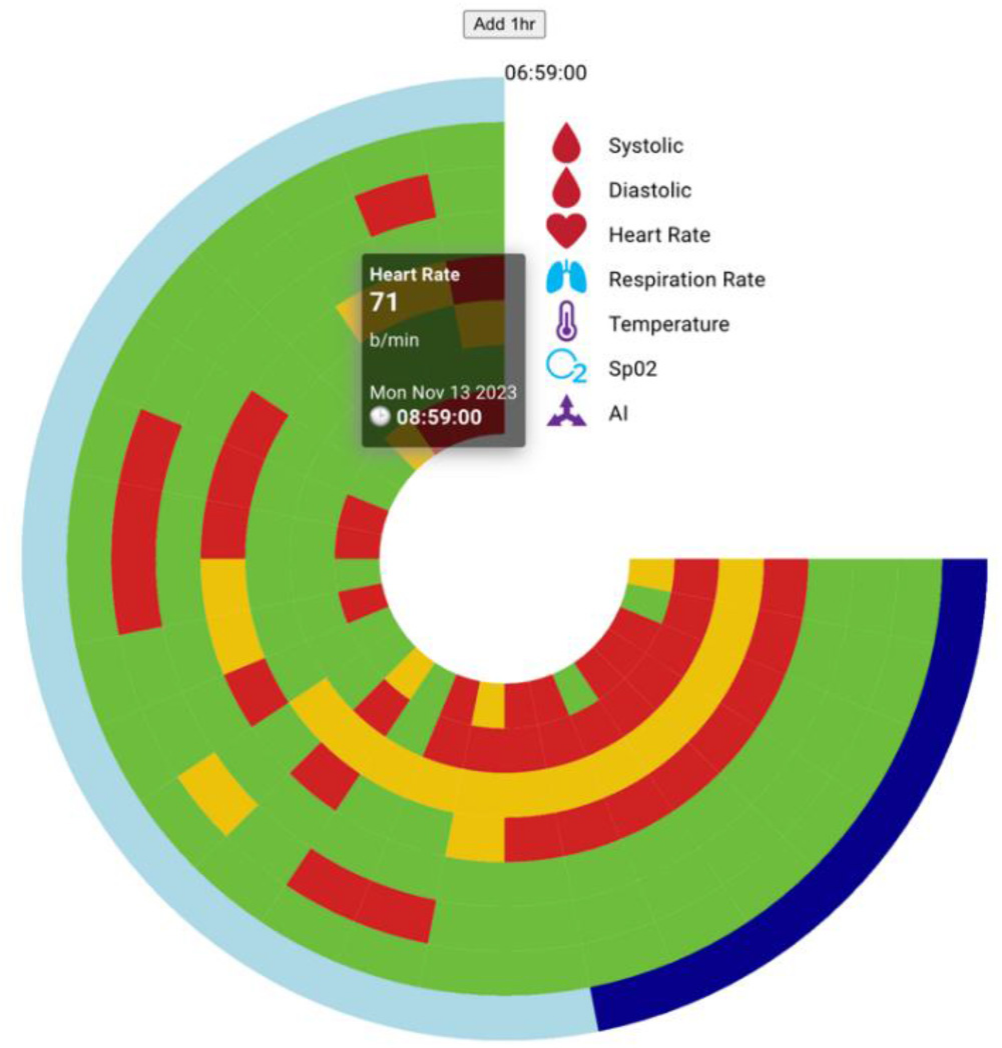
Radial chart for 24-h vital display.

The radial bar chart, depicted in Figure 8, is a novel visualization tool designed to aggregate and display all five vital signs over a 24-h period, aligning with the design criteria specified in requirement R4. This chart showcases the average values for each vital sign at hourly intervals, employing color coded indicators to swiftly communicate the data's status within normal, warning, or danger ranges.

Additionally, this component offers customization options, allowing users to adjust the 24-h viewing window in 1-h increments. This feature significantly enhances the medical practitioner's ability to instantly review and compare the various vital signs at any selected time point with ease. Hovering over the chart reveals the average value for a specific vital sign at a given hour, providing detailed insights at a glance.

A unique aspect of this chart is the inclusion of a day/night indicator on the outermost arc, further enhancing the visual context and understanding of the data in relation to time. This radial bar chart thus stands out as an essential tool for medical practitioners, facilitating a comprehensive and intuitive overview of patient vitals over time.

#### Tab-based visualizations

**Figure 9.**
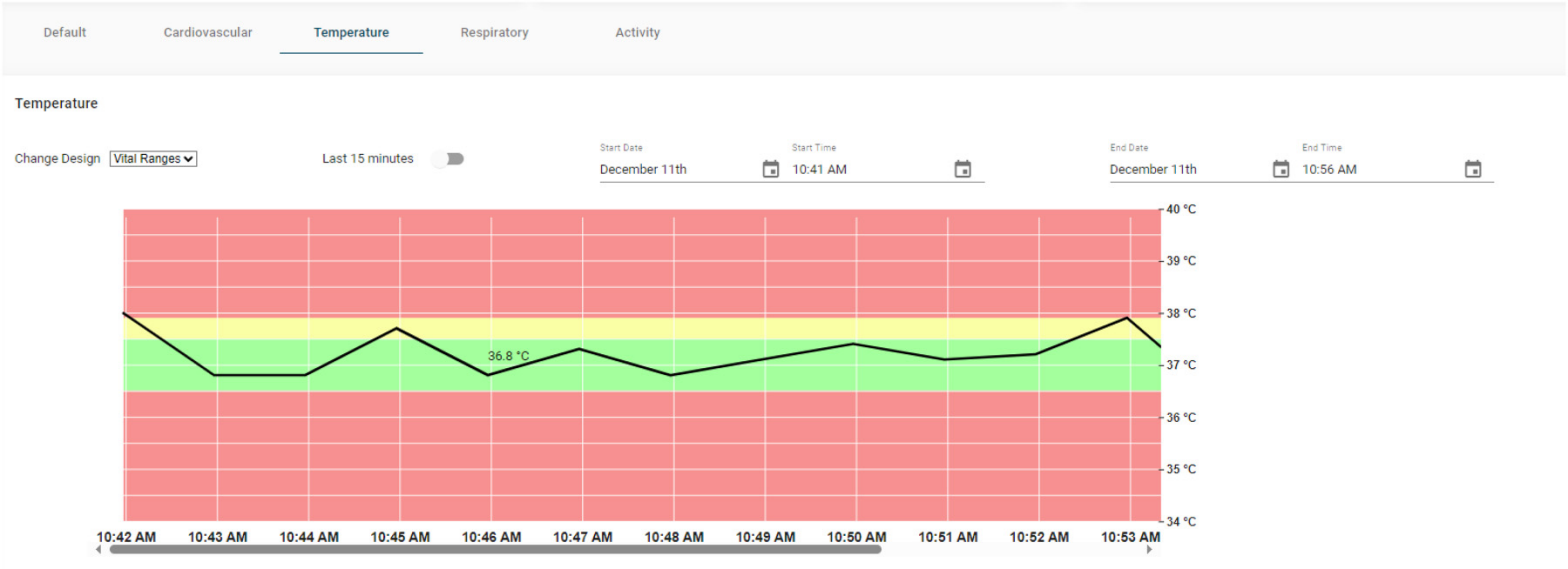
Temperature dashboard.

As described above the default tab in the dashboard has all the components which are displayed in a single page and can be scrolled to view specific data that the user is monitoring. The design has other carefully designed customized views based on the medical condition being monitored for a particular patient. This tab-based approach is highly beneficial for medical practitioners, eliminating the need for extensive scrolling and ensuring that critical information is readily accessible. Moreover, it groups visualizations of related vitals, allowing users to concentrate on specific vitals/signals without overlooking any details. Figure 9 illustrates the Temperature Dashboard, showcasing temperature vital values in detail, exemplifying how the system facilitates focused monitoring of specific health metrics.

As shown in the figure, other tab-based dashboards are:

- the cardiovascular tab focusing on vitals relevant to heart health, including BP, HR, ECG, and current trends for these vitals,
- the Respiratory tab highlighting respiratory rate measurements, and
- the activity tab providing real-time physiological signal values, including data from the accelerometer, PPG, and respiratory waveform, as received from the device.

### Usability study

This section outlines several practical scenarios in which the *RemoteHealthConnect* system could significantly improve patient and caregiver experiences. These scenarios also form the basis of the usability study conducted to assess the system's effectiveness in real-world settings.

Scenario 1: Reporting Dizziness in Early Hours

*Primary Actor*: Medical Doctor

*Goal*: Assist in diagnosing conditions and recommend next steps.

*Pre-Conditions*: A patient who recently underwent a hip replacement and was discharged from the hospital two days ago reports feeling dizzy. The patient is wearing the Vitaliti^TM^ device and calls the nurse line to report the symptoms.

*Steps:*

1. *Initial Review*: The doctor uses her tablet and the *RemoteHealthConnect* app to quickly review various visualizations and radial charts (temperature, SPO2, BP, ECG; see Figure 8: radial chart for 24-h vital display). She checks for any yellow/red sections that might indicate warning/danger ranges for the vitals. Within seconds, she observes that all the patient's vitals fall within the green (safe) region.
2. *Detailed Examination*: Given the patient's history of stage 1 congestive heart failure and current BP medication, the doctor reviews real-time data for temperature, BP, and ECG (see Figure 6: ECG Visualization for Leads 1–3). She also examines the Activity Index to assess the patient's recent activity levels and any potential stress factors that could influence their condition.
3. *Medical Decision*: After analyzing all relevant data, the doctor suspects that one of the medications might be causing the dizziness. She recommends that the patient temporarily discontinue the medication for the next several days to observe if there is any improvement in symptoms.
4. *Follow-Up Plan*: The doctor schedules a check-in with the patient in two days to reassess their condition and specifically inquire about any persistence of dizziness.

Scenario 2: Medical Intervention Using RemoteHealthConnect

*Primary Actor:* Specialist Nurse

*Goal:* Identify and address early warning signs of a potential heart condition to try to keep the patient in their home and avoid hospitalization, if possible.

*Pre-Conditions*: A doctor is remotely monitoring an 86-year-old patient recovering at home from recent hospitalization due to Congestive Heart Failure (CHF). The patient wears the Vitaliti^TM^ device 24/7.

*Steps*:

1. *Initial Observation*: The nurse reviews the patient's 24-h vital sign readings using the radial chart via the *RemoteHealthConnect* app and notes that the HR is increasing and is now in the warning range.
2. *Identifying Symptoms*: Recognizing that this could be an early sign of fluid retention—a common symptom of worsening CHF—the nurse proceeds to check other relevant vitals.
3. *Patient Engagement*: The nurse calls the patient to discuss additional symptoms such as fatigue and shortness of breath. During the conversation, the nurse emphasizes the importance of adhering to prescribed dietary guidelines, including managing fluid and salt intake, monitoring weight, and moderating the use of diuretics like coffee, tea, and caffeinated soft drinks. The patient understands the gravity of the situation and agrees to comply with the dietary restrictions.
4. *Follow-Up and Monitoring*: A 15-min follow-up meeting is scheduled in two days to reassess the patient's condition and ensure compliance with the dietary guidelines.
5. *Outcome*: The use of the web application has facilitated early detection and timely intervention, potentially preventing the escalation of the patient's condition and avoiding hospital readmission.

#### Usability study methodology

We conducted a real-world user experience study divided into two distinct phases. The first phase focused on gathering detailed participant data through a structured survey. This survey aimed to assess the participants’ familiarity with modern technology and their experiences interacting with our web-based application. To enrich our insights, this phase also included researcher observations and the collection of both quantitative data through closed-ended questionnaires and qualitative feedback through open-ended questions and anecdotal comments (see Appendices A and B for the full questionnaire).

Participants for the usability study were recruited using convenience sampling from several distinct groups: applied computer science students, nursing students, IT professionals, and healthcare professionals. This inclusion/exclusion criterion was based on attracting appropriately skilled participants to objectively assess our system from both a UI/UX perspective (computer science and IT professionals) and as a health monitoring tool (nursing students and healthcare professionals).

We introduced the application and its functionalities to nursing students in their classrooms, after which they had the option to complete either a paper or an online survey upon experimenting with the web application. Healthcare professionals were recruited through our established network of healthcare contacts, ensuring a relevant and informed participant base. For computing students, the study was advertised by placing posters on internal communication channels, inviting them to participate and provide feedback on the system's technical aspects. This research was conducted under the Sheridan College Research Ethics Board approval for this study (Approval Number: 2018-12-001-035). All participants involved in this study provided informed consent.

To minimize bias in the survey questionnaire and accurately analyze the performance of our system, we employed the System Usability Scale (SUS) in the second part of our study^39^ (see Appendix C for the System Usability Scale). The SUS has been used in thousands of studies and is widely regarded as both reliable and valid for assessing the usability of various systems and products. In terms of reliability, SUS demonstrates strong internal consistency, with Cronbach's alpha values typically ranging from 0.7 to 0.9.^40,41^ This indicates that the items on the scale are measuring a cohesive construct—usability. Additionally, SUS scores exhibit good test-retest reliability, showing stability over time when the system or product remains unchanged.

Regarding validity, SUS is well-regarded for its construct validity, as it correlates well with other measures of usability, such as expert evaluations and user satisfaction surveys.^41^ This suggests that SUS effectively captures the concept of usability. Criterion validity is also supported by findings that SUS scores align with objective usability measures, such as task performance and error rates, indicating that SUS reflects actual usability in real-world contexts.^41,42^ Furthermore, the scale's content validity is strong, as its items encompass a broad range of usability aspects, including ease of use, complexity, and user satisfaction. This tool provided valuable insights into the usability of our application, allowing us to assess and refine its interface and functionalities based on user feedback.

## Results

The web-based application successfully processes real-time vital data, enabling effective monitoring. Medical practitioners have particularly noted the utility of the customizations available in various visualizations, appreciating the ability to view multiple vital data points through a single component. The color coding in the charts has been instrumental in helping them identify patients with abnormal vital ranges, thereby aiding in prioritizing patient care based on immediate needs.

### Survey results

This section summarizes the survey results derived from “User Information and Demographics” (Appendix A) and “Adaptation and Use” (Appendix B). A total of 30 participants, including nursing and applied computer science students, IT professionals, and healthcare professionals, engaged in the study. Participants were first shown a video demonstration of the *RemoteHealthConnect* app, followed by a live demonstration, after which they were allowed to interact with the app and complete either a paper or an online survey.

Participant Demographics

**Figure 10.**
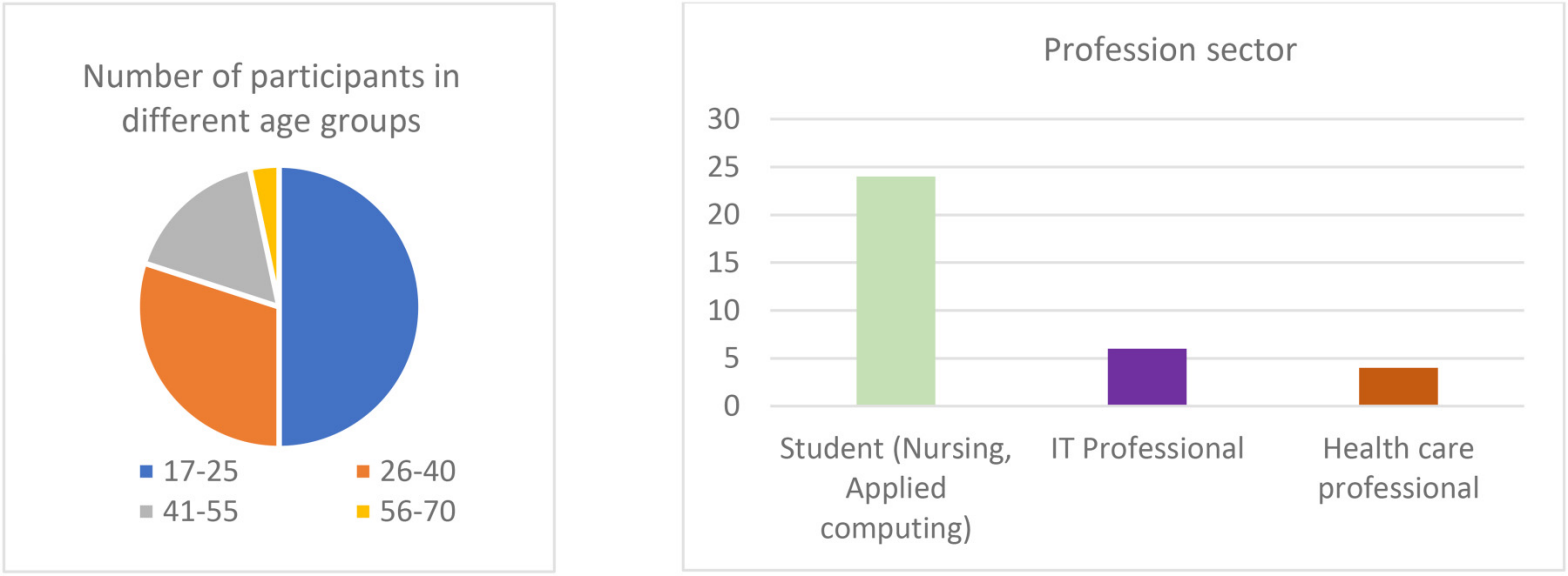
Participant demographics, age groups, and profession.

As shown in Figure 10, about 50% of the participants were students aged 17–25. The remainder were IT and healthcare professionals, reflecting a diverse range of users.

User Feedback on Design and Usability

Feedback on the design and user interface included:

- Visual Design: 63% of participants preferred the Vital Ranges design, which color codes the background for normal, warning, and danger ranges (Question 5, Appendix B).
- Color Coding: 70% gave positive feedback on the color coding scheme used in the visualizations (Question 6, Appendix B).
- Radial Chart: 70% found the radial chart displaying 24-h vital data intuitive and helpful (based on feedback to related survey questions).

Ease of Use, Performance, and Interactivity

From the survey (Questions 2, 8–13, 21, 23; see Appendix B), the findings included:

- 80% of participants rated the usability and performance as meeting or exceeding expectations.
- 93% found the application's user interface straightforward and easy to use without any problems.
- 83% believed the application could significantly impact in-patient care.
- The overall positive impression of *RemoteHealthConnect* was rated at 4.17 out of five, indicating high satisfaction.

Table 1 presents the positive user experience distribution from the usability survey. It shows the distribution of user responses to questions regarding ease of finding and monitoring vital sign information, with ratings from “very hard” to “very easy”). Similarly, Table 2, shows the negative user experience distribution (e.g., user responses to questions about frustration and effort required to use the web application, with ratings from “very high” to “very low”).

**Table 1.** User responses distribution from usability survey representing positive experience.

Questions where higher ratings represent positive experience	User response (%)				
	1 (very low)	2	3	4	5 (very high)
How easy was it to find and monitor the vital sign information that you may be interested in? 1—Very hard, 5—Very easy		3	27	40	30
How easy was it to find and monitor the trends of vital sign data? 1—Very hard, 5—Very easy		3	30	47	20
Did you find everything you wanted on a vital sign monitoring in this web application. 1—Very hard, 5—Very easy		3	27	40	30
Do you think other users would enjoy this web application. 1—No, not at all, 5—Yes, very much			40	40	30

**Table 2.** User responses distribution from usability survey representing negative experience.

Questions where lower ratings represent negative experience	User response (%)				
	1 (very high)	2	3	4	5 (very low)
Frustration—How discouraged, irritated, stressed, or annoyed were you in using this web application? 1—Very high, 5—Very low			13	27	40
Effort—How hard did you have to work to accomplish what you sought out to find in this web application? 1—Worked very hard, 5—Very little work involved			43	27	30

Anecdotal survey responses (Q14 and Q16 in Appendix B)

- Participant 4: “the application would be very useful and helpful for patient care”; “Enjoyed the UI, very intuitive and easy to pick up.”
- Participant 10: “The application is ‘Perfect.’”
- Participant 14: “loved the visuals.”
- Participant 22: “Visuals and color will appeal to most.”

### System usability study results

**Figure 11.**
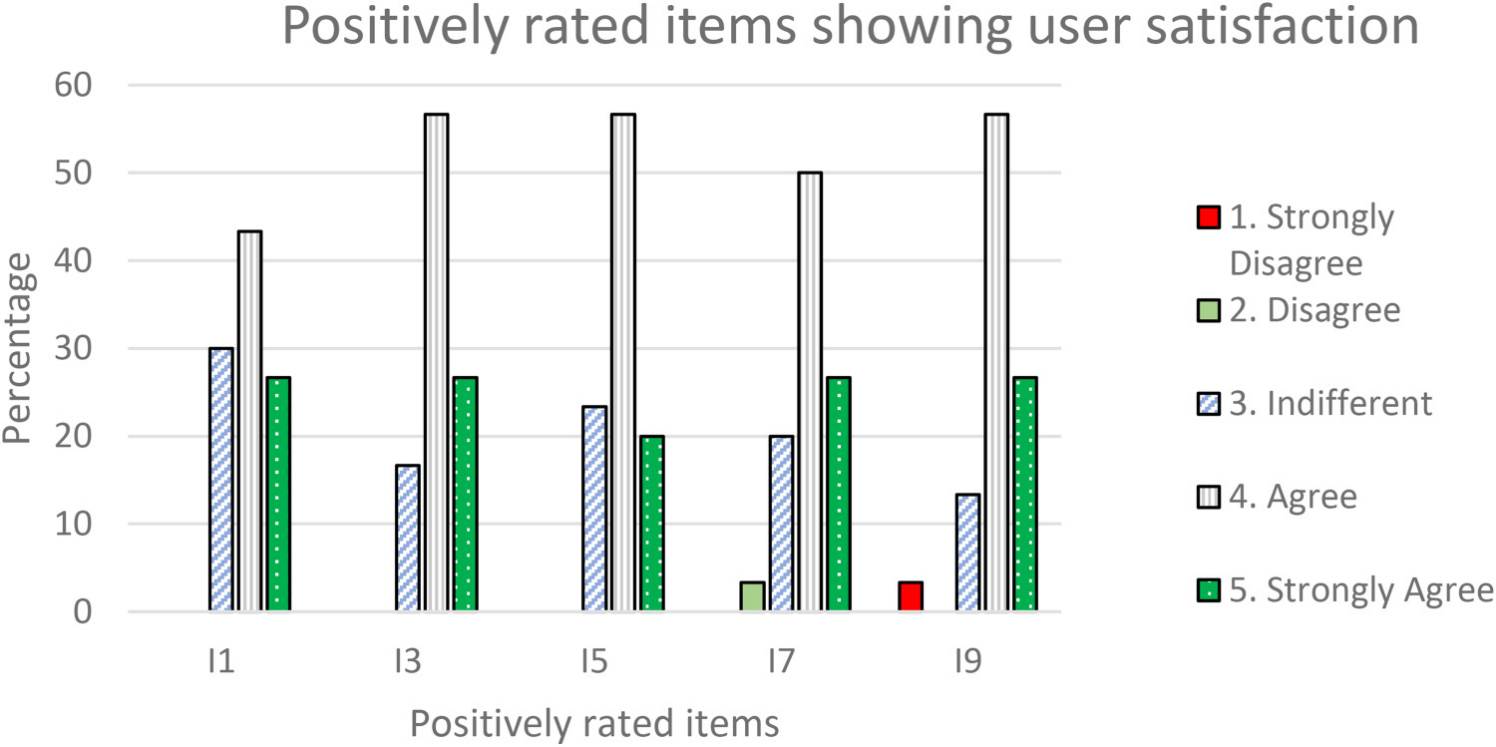
Positively rated items showing user satisfaction.

Quantitative analysis was performed to evaluate *RemoteHealthConnect* with a SUS study.^41^ The SUS odd-numbered items^1^ (I1, I3, I5, I7, and I9) express positive statements on the portal. All of these scored 4 or 5 (“strongly agree” or “agree” with the statement). In total, 70% of the respondents gave scores of 4 or 5 to I1; 83.3% to I3, and I9; and 76.7% to I5 and I7. Figure 11 presents positively rated items showing user satisfaction.

The mean SUS score for this group was 71.50 (min = 50, max = 100, *σ* = 14.38). The average SUS score from 500 studies is 67.5^41^ A one-way ANOVA was performed to determine if there was a difference in the user satisfaction in *RemoteHealthConnect* compared to the SUS average.^43^ There was a statistically significant difference between the groups at the 0.05 level, *F*(1, 58) = 18.208, *p *= .001, showing that the usability of our *RemoteHealthConnect* is above the average of other systems.

**Figure 12.**
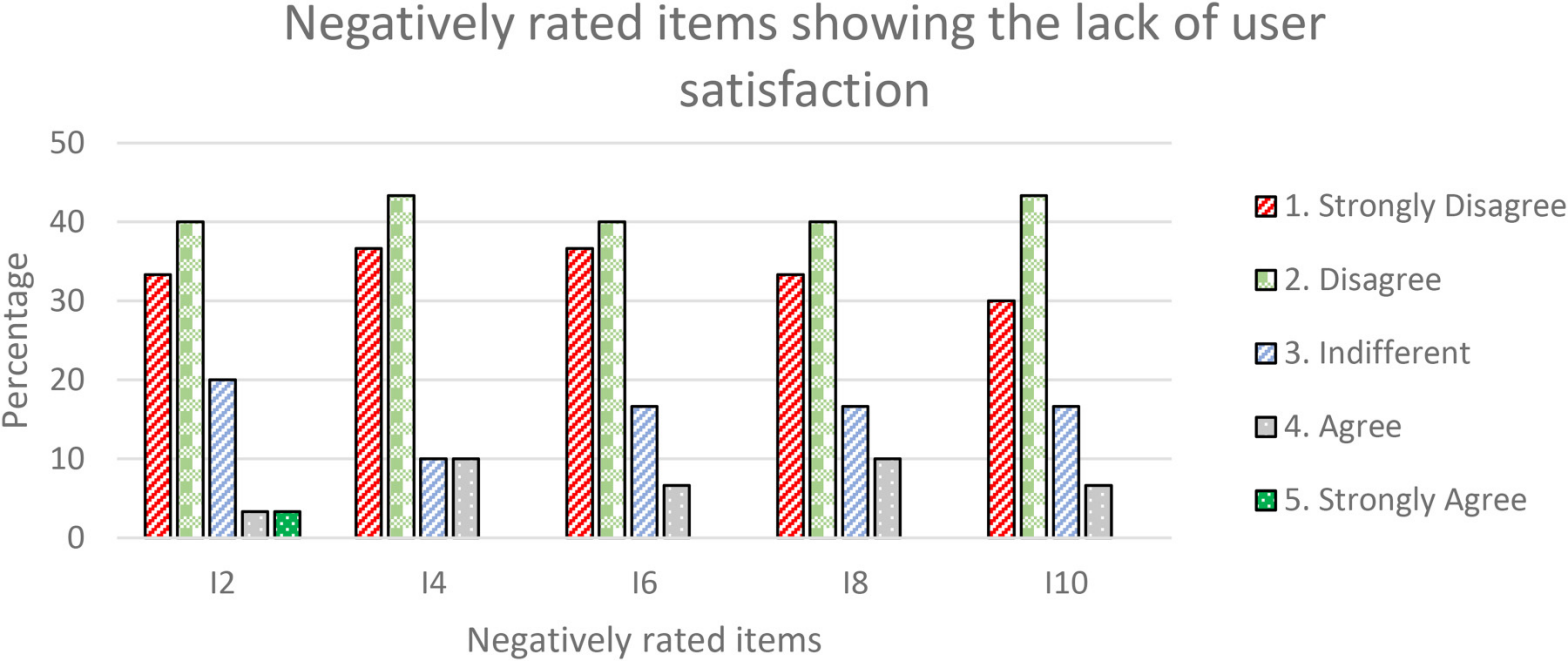
Negatively rated items showing the lack of user satisfaction.

The even-numbered items in the SUS questionnaire^2^ (I2, I4, I6, I8, and I10) express negative statements in using the portal. Most of the respondents gave scores of 1 or 2 (“strongly disagree” or “disagree”) for all items. In total, 73.3% of the respondents gave scores of 1 or 2 to I2, I8, and I10; 80% to I4, and 76.7% to I8. Collectively the responses indicating a high user satisfaction, as shown in Figure 12.

### Summary of survey observations and findings

Throughout the user testing phase, the research team diligently recorded comments, constructive feedback, and observational notes. Based on participants’ responses to the adaptation and use survey questions and the SUS survey, we identified several key findings regarding user comfort and the benefits of using *RemoteHealthConnect*:

1. Interface Customization: Customizing the user interface was highly valued by participants. In the vital trends chart, participants selected all three available designs, demonstrating inclusivity in addressing diverse user preferences. Specifically, 63% preferred Design 1—Vital Ranges, 20% preferred Design 2—Trends, and 17% preferred Design 3—Heatmaps.
2. Positive Responses to Design Requirements: Approximately 70% of participants responded positively to the main research requirements—namely R2: Selection of Visualization Types, R3: Incorporating Interactivity and Customization, and R4: 24-h Vital Data Display Component for Multiple Vitals. This feedback suggests that the design successfully resonated with end-users.
3. Ease of Navigation: 80% of participants appreciated the ease of navigating the application and were able to effectively find and monitor the vital data they were interested in.
4. Color Coding: The intuitive use of color coding was highlighted by 70% of the participants, with one participant noting, “Visual and colour will appeal to most,” which underscores the effectiveness of this design choice.
5. Overall Positive Experience: As shown in Table 1, nearly 70% of participants had a positive experience, rating their satisfaction as high or very high regarding the usability aspects queried.
6. Absence of Negative Experience: Table 2 reveals that participants reported no negative experiences while using the application, indicating a successful user interface and interaction design.

## Discussion

The design of the *RemoteHealthConnect* system extends well beyond its initial application in post-operative care. Although the web-based application is accessible from various devices, user feedback indicates that tablets, laptops, and desktops provide the optimal experience due to their larger screens, which facilitate clearer views, multitasking capabilities, and higher resolutions that make it easier to discern trends and data variations.

The system is crafted to be intuitive, adhering to recommended design protocols. Plans are underway to further reduce the learning curve by integrating comprehensive tutorials and user guides, enabling users to tailor their experience according to individual preferences.

However, the true potential of *RemoteHealthConnect* extends beyond post-operative care. Its functionalities could significantly enhance other healthcare settings, such as ambulance services. For example, real-time transmission of patient vitals from ambulances directly to hospital emergency departments could improve triage and patient preparation upon arrival. An illustrative scenario shared by a medical expert involved a doctor using the system at a nursing station to prioritize patient care. These scenarios underscore the versatility and potential of *RemoteHealthConnect* to transcend its original use, offering valuable tools for enhanced RPM across various medical contexts.

To objectively assess its impact, we propose a follow-up study with a control group. Participants will be divided into two groups: those using *RemoteHealthConnect* and those employing traditional monitoring practices. The Post-Study System Usability Questionnaire (PSSUQ) will evaluate user experience across various aspects. By comparing PSSUQ scores between the groups, we aim to demonstrate whether our system offers a more user-friendly and efficient approach. This control group study will provide data-driven evidence of the system's effectiveness and guide future iterations for continuous improvement, highlighting *RemoteHealthConnect's* potential to revolutionize RPM practices.

## Conclusion

Our research focused on developing *RemoteHealthConnect*, a user-centric RPM application designed specifically to meet the needs of healthcare professionals. We achieved significant advancements by integrating a series of innovative features that enhance both the user experience and the effectiveness of patient monitoring. These features include customizable dashboards, user-defined data range selections, diverse visualization options, real-time radial charts, and color-coded data representations.

The incorporation of these functionalities has led to the creation of an application that is not only user-friendly but also highly informative. *RemoteHealthConnect* empowers healthcare professionals with essential tools to monitor patients more efficiently, make informed decisions quickly, and ultimately enhance patient care. The principles of user-centric design applied in our project provide a robust foundation for future developments in RPM applications. This approach ensures that future technologies will continue to improve patient outcomes and contribute to a more effective healthcare system.

A video demonstration of the *RemoteHealthConnect* system is available on YouTube via the following link. The video provides a comprehensive review of all the visualizations and user interface interactions discussed in this paper.

### Limitations

The *RemoteHealthConnect* system holds significant promise for advancing RPM. Despite its potential, it is important to recognize certain limitations that provide avenues for future enhancements. The system processes a massive volume of raw data, specifically 1000 data points per second per signal. To manage this effectively, the data handling layer currently downsamples this data before transmission to the backend server. While this approach facilitates real-time visualization, further exploration into advanced data compression techniques could reduce information loss significantly.

Another critical issue is the system's dependence on stable network connections. Network interruptions can lead to data losses, albeit temporarily mitigated by the sensor device's onboard storage, which caches vital data for up to a week during outages. Once connectivity is restored, the application retrieves this offline data, though this results in temporary interruptions in real-time data display, currently indicated by greyed-out portions on the app. Future iterations could benefit from exploring seamless data buffering techniques and improved display methods during network fluctuations.

Furthermore, the participant diversity in our usability study skewed towards computing students, potentially limiting the generalizability of the findings. Future studies should aim for a more balanced participant pool, with a larger representation of nursing and medical professionals to better reflect the target user groups. Additionally, the system's performance with multiple concurrent users remains untested, necessitating backend server enhancements to ensure scalability as user numbers increase.

### Future research

Looking forward, the *RemoteHealthConnect* system is designed with adaptability in mind, aiming to provide a generic platform suitable for monitoring a wide range of health conditions. Several exciting enhancements are planned to further this capability. For instance, future iterations will enable medical professionals to customize monitoring windows for each vital sign based on individual patient needs, such as setting different observation durations for temperature trends versus BP.

Moreover, the addition of multi-patient management capabilities will allow physicians to efficiently monitor larger groups of patients by seamlessly switching between multiple patient profiles. As the accumulation of sensor data grows, the integration of big data technologies will become crucial. This will not only streamline data analysis and storage but also enable the use of data mining techniques to uncover hidden patterns within the data, potentially leading to novel insights.

Patient data privacy remains a paramount concern, and ongoing efforts to integrate standards like HIPAA and PIPEDA are critical in ensuring the highest level of data security and patient privacy. Additionally, plans include integrating the *RemoteHealthConnect* system with the Hamilton Early Warning Score (HEWS),^44^ which uses calculated scores from vital signs to facilitate early detection of subtle changes in patient health, potentially enabling earlier interventions and improving patient outcomes.

These future directions not only aim to enhance the functionality and effectiveness of *RemoteHealthConnect* but also highlight its potential to revolutionize RPM across various healthcare settings.

## Notes

1. SUS positive rated items: Item I1: “I think that I would like to use this system frequently” Item I3: “I thought the system was easy to use,” Item I5: “I found the various functions in this system were well integrated” Item I7 “I would imagine that most people would learn to use this system very quickly” Item I9: “I felt very confident using the system.”

2. SUS negative rated items: Item I2: “I found the system unnecessarily complex” Item I4: “I think that I would need the support of a technical person to be able to use this system,” Item I6: “I thought there was too much inconsistency in this system” Item I8 “I found the system very cumbersome to use” Item I10: “I needed to learn a lot of things before I could get going with this system.”

## Appendix A

User information and demographics

1. What is your full name? (optional)
2. What is your email? (optional)
3. What is your age group?
  - 17–25
  - 26–40
  - 41–55
  - 56–70
  - Prefer not to answer.
4. How do you describe your gender
  - Female
  - Male
  - Prefer not to say.
  - Other
5. What is your professional sector (Please select multiple if appropriate)
  Student
  - IT Professional (e.g., faculty, researcher, project manager, developer, etc.)
  - Healthcare /Patient care professional (e.g., physician, nurse, physiotherapist, etc.)
  - Healthcare application development (e.g., software designer/developer, specialized healthcare)
  - Administration/office management (e.g., office staff, admin staff)
  - Other, please specify.
6. How often do you use electronic devices (e.g., smartphones, tablets, laptops, etc.)
  - Often—several times a day
  - Couple times a day.
  - Couple times a week.
  - Occasionally
  - Never

## Appendix B

Adaptation and Use

7. On a scale of 1 to 5, how easy was it to navigate through the web application (1 = very difficult, 5 = very easy)
8. Did you encounter any issues with the application's user interface or design?
9. If you answered “Yes” to the previous question, please specify the issues you encountered.
10. How would you rate the responsiveness and speed of the web application?
  - Very slow
  - Slow
  - Average
  - Fast
  - Very fast
11. Of the 3 Blood pressure visualizations, which one do you think best appeals to you (in terms of data clarity, usefulness)
  - Vital ranges
  - Trends
  - Heatmap
  - Its all the same
12. What do you think about the color coding used in the radial graph (Green—normal, Yellow- warning, Red—danger)
  - Love it, its very intuitive.
  - Like it, its easy to understand.
  - Neutral, it doesn’t make a significant difference.
  - Somewhat dislike it, buts its manageable
  - Dislike it, its confusing.
  - Don’t have an opinion.
13. What do you think about having 24-h data for all vitals represented in a single radial chart?
  - Love it, its very intuitive.
  - Like it, its easy to understand.
  - Neutral, it doesn’t make a significant difference.
  - Somewhat dislike it, buts its manageable
  - Dislike it, its confusing.
  - Don’t have an opinion.
14. How easy was it to find and monitor the vital sign information that you may be interested in?
  1—Very hard,      5—Very easy
  1  2  3  4  5
15. How easy was it to find and monitor the trends of vital sign data
  1—Very hard,      5—Very easy
  1  2  3  4  5
16. Did you find everything you wanted on a vital sign monitoring in this web application,
  1—Very hard,      5—Very easy
  1  2  3  4  5
17. Effort—How hard did you have to work to accomplish what you sought out to find in this web application?
  1—Very hard,      5—Very easy
  1  2  3  4  5
18. Frustration—How discouraged, irritated, stressed, or annoyed were you in using this web application?
  1—Very high,      5—Very low
  1  2  3  4  5
19. Do you think other users would enjoy this web application?
  1—No, not at all      5—Yes, very much
  1  2  3  4  5
20. Please share any additional comments regarding this study (if any)
21. How impactful/helpful the web application would be in patient care?
  - Insignificant
  - Somewhat significant
  - Significant
  - Very Significant
22. Please add any comments on the answer to the previous question
23. Please rate your overall experience with us
  1—Not satisfied      5—Very satisfied
  1  2  3  4  5

## Appendix C

System Usability Scale

**Table:**

	Strongly disagree Strongly agree
I think that I would like to use this system frequently I found the system unnecessarily complex I thought the system was easy to use I think that I would need the support of a technical person to be able to use this system I found the various functions in this system were well integrated I thought there was too much inconsistency in this system I would imagine that most people would learn to use this system very quickly I found the system very cumbersome to use I felt very confident using the system I needed to learn a lot of things before I could get going with this system	
